# Modelling COVID-19 growth cases of provinces in java Island by modified spatial weight matrix GSTAR through railroad passenger's mobility

**DOI:** 10.1016/j.heliyon.2021.e06025

**Published:** 2021-02-02

**Authors:** U.S. Pasaribu, U. Mukhaiyar, N.M. Huda, K.N. Sari, S.W. Indratno

**Affiliations:** Statistics Research Division, Faculty of Mathematics and Natural Sciences, Institut Teknologi Bandung, Indonesia

**Keywords:** Weight matrix, Distance, Train, 2019-nCoV, GSTAR(1;1)

## Abstract

The movement of positive people Coronavirus Disease that was discovered in 2019 (Covid-19), written 2019-nCoV, from one location to another has a great opportunity to transmit the virus to more people. High-risk locations for transmission of the virus are public transportations, one of which is the train, because many people take turns in or together inside. One of the policies of the government is physical distancing, then followed by large-scale social restrictions. The keys to the policy are distance and movement. The most famous transportation used for the movement of people among provinces on Java is train. Here a Generalized Space Time Autoregressive (GSTAR) model is applied to forecast infected case of 2019-nCoV for 6 provinces in Java. The specialty of this model is the weight matrix as a tool to see spatial dependence. Here, the modified Inverse Distance Weight matrix is proposed as a combination of the population ratio factor with the average distance of an inter-provincial train on the island of Java. The GSTAR model (1; 1) can capture the pattern of daily cases increase in 2019-nCoV, evidenced by representative results, especially in East Java, where the increase in cases is strongly influenced by other provinces on the island of Java. Based on the Mean Squares of Residuals, it is obtained that the modified matrix gives better result in both estimating (in-sample) and forecasting (out-sample) compare with the ordinary matrix.

## 1 Introduction

The world now has been fighting with the big problem, which not only has a massive effect on the health sector but also almost all sectors in life, that is, pandemic coronavirus disease. On December, 31^st^ 2019, the World Health Organization's (WHO) China office heard the first reports of a previously-unknown virus behind some pneumonia cases in Wuhan, a city in Eastern China (WHO, 2020). What started as an epidemic mainly limited to China has become a truly global pandemic start on March 11^st^, 2020. The 2019-nCoV is an infectious disease caused by a newly discovered coronavirus (WHO, 2020). This virus is transmitted between people through respiratory droplets when symptomatic people speak, sneeze, or cough (Ningthoujam Ramananda, 2020). Droplets can land on the mouth, nose or eyes of the people at a close range (CDC, 2020). Virus particles that have small enough considered as aerosols or fine particles can remain suspended in the air for hours and can walk with water currents across tens of feet. Furthermore, 2019-nCoV is more stable in plastic and stainless steel in copper and cardboard, and can survive up to 72 h after application to this surface (Doramelen et al., 2020). Viral particles can also be emitted from the surface, such as toilets, transportations, and on surfaces that are frequently touched (Santarpia et al., 2020). People may become infected if they touch their face after touching a surface that had been contaminated by the virus. The amount of virus declines overtime on the surface for hours or days. Regarding the ways in transmitting the virus, the CDC recommends maintaining at least a six-foot distance among people (CDC, 2020). Due to the gravity, those large droplets (which are more prominent than about .0002 inches, in size) fall into the ground within a distance of six feet from the infected person (Live Science, 2020). The public transportation sector is a prime agent for spreading the 2019-nCoV. Therefore, the interaction carried out between passengers is a rapid medium for 2019-nCoV virus transmission.

On March, 2^nd^ 2020, the 2019-nCoV had entered Indonesia (Kementerian Luar Negeri, 2020). The virus has infected two residents of Depok, West Java. As of June 11^th^, 2020, the Ministry of Health recorded 35,295 positive cases in Indonesia, with 12,636 people recovering and 2,000 dead (BNPB, 2020). The number of 2019-nCoV cases in Indonesia continues to increase, especially in Java. This island can be categorized to be the epicenter of the 2019-nCoV case, especially in the province of DKI Jakarta (8,650 positive cases) and now the addition of the most daily cases occurred in East Java since the end of May (the highest on May 21^st^ of 502 new positive cases). The increasing positive cases are caused by many factors, including (1) high mobility between provinces in Java, (2) DKI Jakarta as the capital city of Indonesia, and (3) many migrants from provinces other than DKI Jakarta on the Java's island who work or live in DKI Jakarta, thus allowing the movement of people from one province to another province on Java. This movement has a significant probability of transmitting the 2019-nCoV virus. One of the places that are susceptible to the virus transmission is public transportations. Several factors increase the potential risk of 2019-nCoV transmission in public transportation: the crowd, the air ventilation inside, and the amount of time spent on public transportation. Based on the Ministry of Transportation's monitoring, during December 2019 to January 2020, the transportations widely used in Java's island are 34% Train, 30% Plane, 16% Bus, 6% Sea transportation, and 14% private vehicle and ferry (Departemen Perhubungan, 2020). According to DKI Jakarta Provincial Government, the train routes within Jabodetabek (Jakarta, Bogor, Depok, Tangerang, and Bekasi) area and from Bogor/Depok to Jakarta City/Angke/Jatinegara, have the highest risk in transmitting the virus, since they serve more than 500,000 people per day. Those routes pass three provinces (Banten, DKI Jakarta, and West Java) simultaneously. Therefore, it is urgently to explore train mobility in modeling the transmission of the 2019-nCoV virus in Java.

The distribution of 2019-nCoV cases among provinces in Java can be analyzed through the Generalized Space Time Autoregressive, stated as GSTAR (1; 1) model. The model assumes that events at a location are not only influenced by past events but also influenced by past events at neighboring locations (Mukhaiyar and Pasaribu, 2012). One uniqueness of this model is the weight matrix representing the spatial dependence of an event in a location with its surrounding location. Here, a modified Inverse Distance Weight, abbreviated as IDW, matrix is proposed that representing the spatial dependency of the distance of a train among provinces in analyzing the transmission of the 2019-nCoV case in Java. The object used is the daily positive cases of 2019-nCoV in six provinces of Java island, i.e Banten, DKI Jakarta, West Java, Central Java, DI Yogyakarta (DIY), and East Java. Then the purpose is to predict 2019-nCoV increment cases Java based on the Spatio-temporal GSTAR (1; 1) model.

Researchers are working intensively on 2019-nCoV. From the dynamic side, Li, et al. (2020) and Kucharski et al. (2020) about early transmission dynamics in Wuhan. The individuals have classified into four compartments classes, as follows: susceptible, exposed (but not yet infectious), infectious, and removed (i.e, isolated, recovered, or otherwise no longer infectious). Therefore, a SEIR transmission model is chosen in this case. The model can reproduce the observed temporal trend of cases within Wuhan and cases exported internationally. It also captures the exponential growth in case onsets in early January, the rising number of exported case onsets between Jan 15^th^ and 23^rd^, 2020, and the prevalence of infection measured on ten evacuation flights from Wuhan to seven countries. The prediction of 2019-nCoV cases using statistical models is also developing rapidly at this time. An SEIR is also chosen for modeling the virus transmission in Wuhan, Diamond Princess, and Jakarta-cluster (Soewono, 2020). Tandon et al. (2020) used the ARIMA model to predict 2019-nCoV cases in India. While Abdulmajeed et al. (2020) tried to compare the ARMA model, the prophet (time-series regression forecasting) and Holt Winter Exponential smoothing to predict 2019-nCoV cases in Nigeria. Modification of the ARIMA model by adopting a hybrid model was also carried out by Chakraborty and Ghosh (2020) to predict 2019-nCoV cases in several countries, namely Canada, France, India, South Korea, and the UK. The Generalized Logistic growth Model (GLM) can also be used to generate short-term forecasts in real-time in China (Roosa et al., 2020). The susceptible-exposed-infectious-recovered model was also used by Wu et al. (2020) to predict the potential domestic and international spread of the 2019-nCov outbreak originating in Wuhan.

This paper is divided into four sections. The first section gives an introduction of 2019-nCoV. Section II briefly explains the modified IDW matrix, and it is followed by data analysis using GSTAR(1; 1) as outlined in section III. Conclusions and remarks are put forward in the fourth section.

## 2 GSTAR with modified inverse distance – spatial weight matrix

Let follows the GSTAR(1; 1) model,

(1) $$Z_{t}={\text{Φ}}_{10}Z_{t-1}+{\text{Φ}}_{11}WZ_{t-1}+e_{t}$$

where $Z_{t}$ is stationary data at time t, ${\text{Φ}}_{10}$ is the diagonal matrix of autoregressive parameters for first lag of time and zero lag of spatial order, while ${\text{Φ}}_{11}$ is the diagonal matrix of autoregressive parameters for first lag of time and first lag of spatial order, $e_{t}$ is a noise process at time t, and $W=\left[w_{ij}\right]$ is a matrix, called spatial weight matrix for location j to i. This matrix is the beauty of the GSTAR model, since it represents the spatial dependency between locations.

Some development of the GSTAR (1; 1) has been done by some researchers, such as making a new procedure for Generalized STAR modeling using IAcM (Inverse Autocovariance Matrix) approach (Mukhaiyar and Pasaribu, 2012), considering an exogenous variable to the model (Huda, 2019) and also outlier factor (Mukhaiyar et al., 2020). This model was applied to the monthly tea production of some plantations in West Java, Indonesia. In terms of weighting on the GSTAR, GSTAR has been modeled using the weighted average of the fuzzy sets concept approach and applied that model to oil palm production (Nugraha et al., 2015). Yundari et al. (2017) researched error assumptions on the GSTAR. Recently Yundari et al. (2018) researched the Spatial Weight Determination of GSTAR(1; 1) by using kernel function. This research made that weight matrix construction was less subjective. In application, the GSTAR is rapidly used to forecast Gross Domestic Product (GDP) West European (Nurhayati et al., 2012), chili price in Bandung's market Fadlilah (2015), and criminality (Masteriana and Mukhaiyar, 2019). The combination of GSTAR modeling and variogram of spatial analysis was conducted by Mukhaiyar (2015). Sari et al. (2015) use a bootstrap approach to estimate the parameters of isotropic semivariogram, while Permai et al. (2018) use a spatial weighting approach to disaggregate Millennium Development Goals (MDGs) indicators. Furthermore, the effect of spatial aggregation on the space-time model was investigated by Gehman (2016). The latest research is GSTAR model for discrete random variables (Huda et al., 2021)

The focus in this paper is the weight matrix. Generally, researchers use uniform weights (Nurhayati et al., 2012), binary (Mukhaiyar and Pasaribu, 2012), or non-uniform weights based on distance. This weight selection process is still subjective. The proposed weight matrix also assumes the closer the distance, the stronger the relationship between provinces. This matrix is a combination of the ordinary IDW matrix with the ratio of populations between provinces and named as modified IDW matrix. Note that one of the factors affecting the transmission of 2019-nCoV on Java's island is the large number of people moving from one province to another. One of the leading causes is the location of the person's work. Therefore, the population ratio also plays an important role in the transmission of 2019-nCoV cases.

Let $W^{*}={\left[w_{ij}^{*}\right]}_{N\times N}$ is the modified IDW matrix, where N is the number of locations used. The $w_{ij}^{*}$ is the result of normalization from $w_{ij}^{\left(\ell \right)}\cdot r_{ij}$, where $r_{ij}$ is the ratio of populations from location j to i and $w_{ij}^{\left(\ell \right)}$ is the entry of $i^{th}-$ row and $j^{th}-$ column of the ordinary IDW matrix W. The weight calculation is obtained from the normalization of the actual inverse distance result. In general, the ordinary IDW for each location is expressed by

(2) wij(ℓ)=1dij∑j=1N1dij

where i≠j, the total weight for each location is 1 $\left(\overset{N}{\underset{j=1}{\sum }}w_{ij}^{\left(\ell \right)}=1\right)$ and for all locations is $N\;\left(\overset{N}{\underset{i=1}{\sum }}\overset{N}{\underset{j=1}{\sum }}w_{ij}^{\left(\ell \right)}=N\right)$, $d_{ij}$ is average distance among the provinces (i and j) based on the average track length between pairs of train routes (see Table 1).

**Table 1:** Average distance among the provinces based on the average track length between pairs of train routes (see Figure 1). The distance between provinces obtained is not symmetrical because not all trains have an alternating route.

Distance $\left(d_{\mathrm{ij}}\right)$	Banten	DKI Jakarta	West Java	Central Java	DIY	East Java
Banten	0	41.30	139.87	553.30	571.30	847.70
DKI Jakarta	41.30	0	98.57	512.00	530.00	806.40
West Java	262.80	221.50	0	448.00	382.30	717.25
Central Java	494.68	453.38	448.00	0	66.00	592.50
DIY	571.30	530.00	382.30	66.00	0	352.00
East Java	847.70	806.40	717.25	592.50	352.00	0

**Figure 1:**
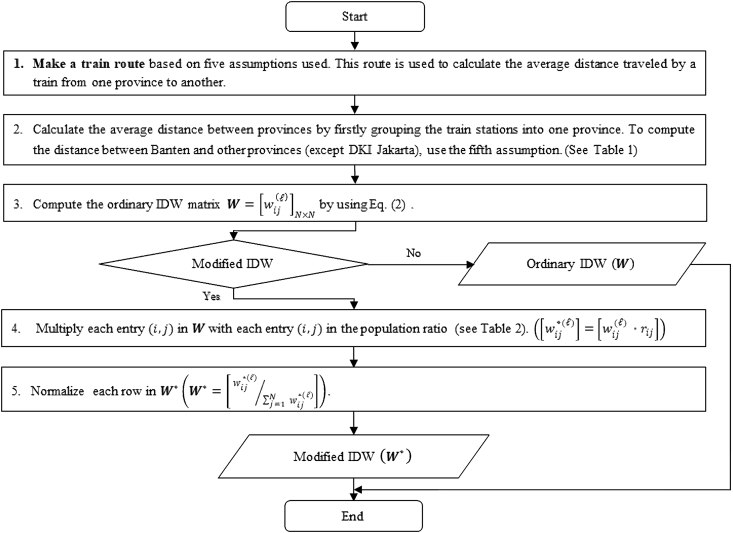
Flowchart of computing the modified IDW matrix. The train route can be seen in Figure 2.

The procedure for defining the modified IDW matrix in the provinces of Java was shown in Figure 1. In the making of the train route, the following assumptions are used.

1. The transportation mode used is only the train with a mixed-class carriage type.
2. There are two kinds of trains be observed, (1) Electric train (30 trains), and (2) Commuter lines (Banten, DKI Jakarta, West Java),
3. There are 33 train stations (t.s) involved, consisting of Banten (5 t.s), DKI Jakarta (7 t.s), West Java (9 t.s), Central Java (5 t.s), DIY (2 t.s), and East Java (5 t.s),
4. The route used is the electric train and commuter line (listed in point 3) by only taking the initial and final stations. If multiple routes have the same initial and final stations, then the route with the closest distance is chosen,
5. If there is no electric train/commuter line connecting the two provinces, say A to B, then it assumes that the people will go to another province, say C, firstly with an electric train/commuter line to connect them. The province C chosen was the province with the shortest distance to that province B.

The modified IDW matrix can be defined as,

$$W^{*}={\left[\frac{w_{ij}\cdot r_{ij}}{\sum_{j=1}^{N}\left(w_{ij}\cdot r_{ij}\right)}\right]}_{N\times N}$$

where

$w_{ij}$ is entry of ordinary IDW matrix W (obtained from Eq. (2) and the third step in Figure 3) and $r_{ij}$ is population ratio for location j to i (see Table 2).

**Figure 2:**
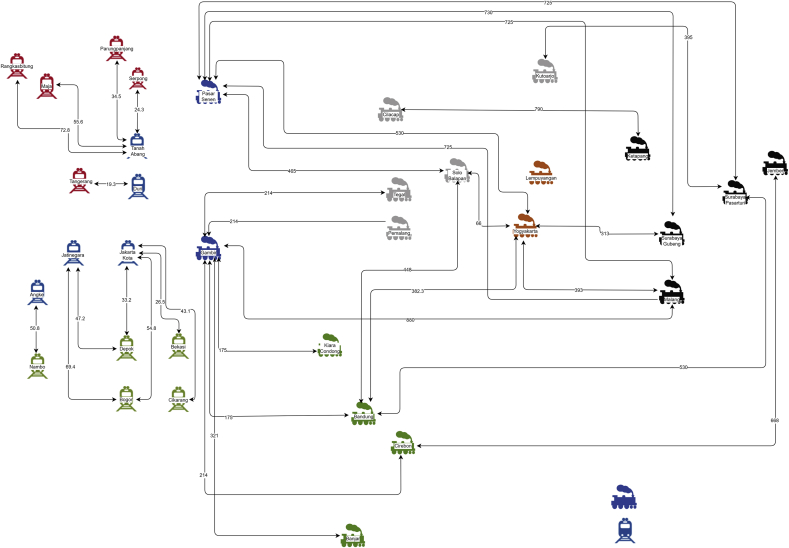
The train routes among provinces in Java based on five assumptions.

**Figure 3:**
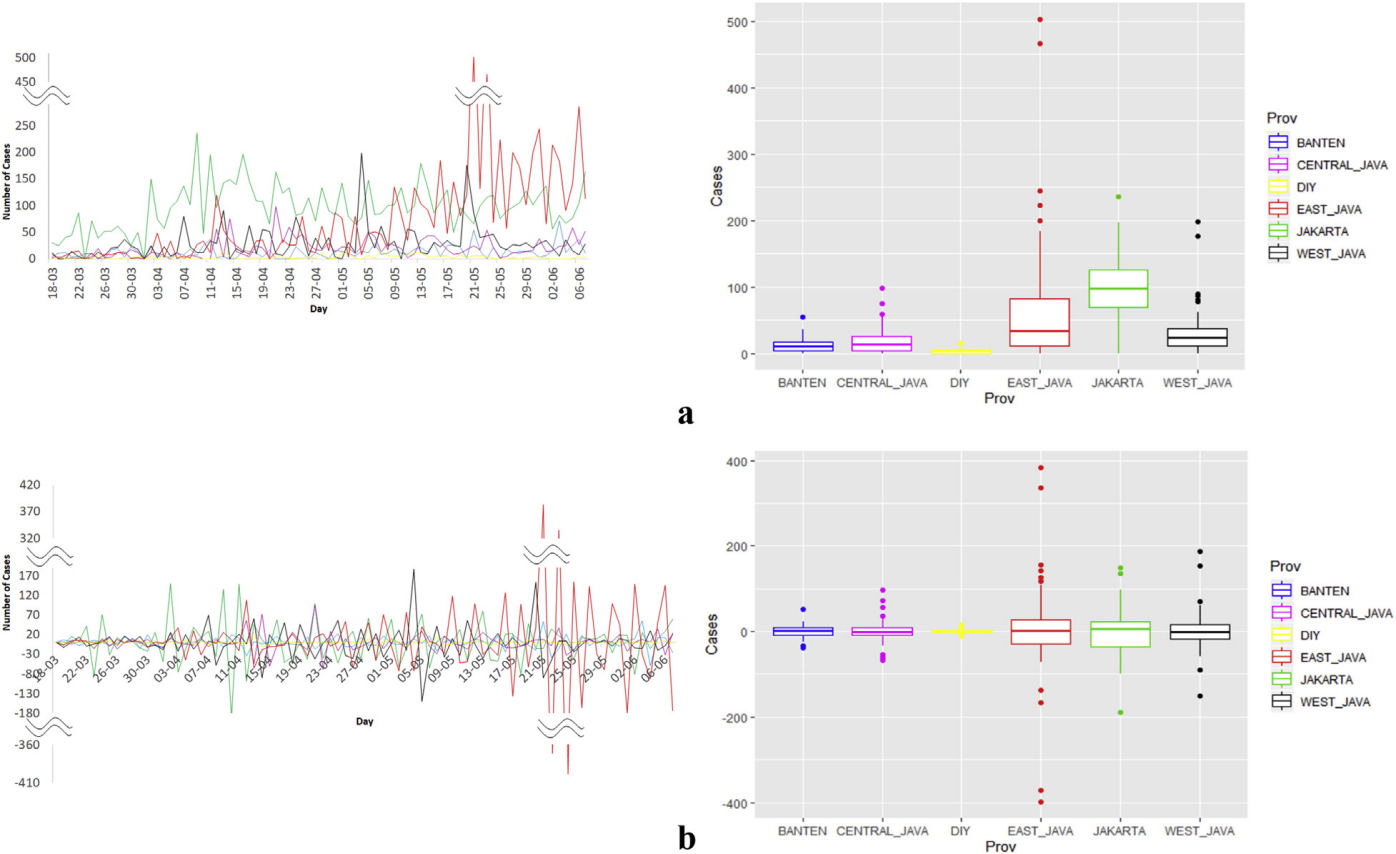
Time series plot and boxplot of daily cases 2019-nCoV (a) without differencing and (b) after first differencing. Differentiation makes more outliers detected. Since May 17^th^, 2020, the increase in daily 2019-nCoV cases in East Java has exceeded DKI Jakarta, which is the epicenter of 2019-nCoV.

**Table 2:** Population ratio among the provinces. The writing in bracket is total population in a province. The West Java Province is the largest population, followed by East Java and Central Java.

Ratio $\left(r_{ij}\right)$	Banten (12,927,316)	DKI Jakarta (10,467,629)	West Java (49,316,712)	Central Java (34,490,835)	DIY (3,842,932)	East Java (39,292,972)
Banten	1.000	0.810	3.815	2.668	0.297	3.039
DKI Jakarta	1.235	1.000	4.711	3.295	0.367	3.754
West Java	0.262	0.212	1.000	0.699	0.078	0.797
Central Java	0.375	0.303	1.430	1.000	0.111	0.139
DIY	3.364	2.724	12.833	8.975	1.000	10.225
East Java	0.329	0.266	1.255	0.878	0.098	1.000

For example, computing the IDW from DKI Jakarta to Banten.

$$w_{12}^{*(1)}=\frac{w_{12}^{\left(1\right)}.r_{12}}{\left(w_{12}^{\left(1\right)}.r_{12}\right)+(w_{13}^{\left(1\right)}.r_{13})}$$

where w12(1)=1d121d12+1d13=141.3141.30+1139.87=0.772, w13(1)=1d131d12+1d13=1139,87141.30+1139.87=0.228, $r_{12}\;$ and $r_{13}$ are in Table 2.

Then the $w_{12}^{*\left(1\right)}$ is defined as

$$w_{12}^{*\left(1\right)}=\frac{0.772\cdot 0.810}{\left(0.772\cdot 0.810\right)+\left(0.228\cdot 3.815\right)}=\frac{0.625}{1.495}=0.418$$

and using the same way, each element of the modified IDW matrix can be obtained, i.e.

(3) $$W^{*\left(1\right)}=10^{-3}\cdot \left[\begin{array}{ll} W_{11}^{*\left(1\right)}\; & O\\ O & W_{22}^{*\left(1\right)} \end{array}\right],\;W^{*\left(2\right)}=10^{-3}\cdot \left[\begin{array}{ll} \;O & W_{12}^{*\left(2\right)}\\ W_{21}^{*\left(2\right)} & W_{22}^{*\left(2\right)} \end{array}\right],\;and\;W^{*\left(3\right)}=10^{-3}\cdot \left[\begin{array}{ll} \;O & W_{12}^{*\left(3\right)}\\ W_{21}^{*\left(3\right)} & W_{22}^{*\left(3\right)} \end{array}\right]$$

where

$W_{11}^{*\left(1\right)}=\left[\begin{array}{lll} \;0\; & 418 & 582\\ 384 & \;0\; & 615\\ 510 & 490 & \;0\; \end{array}\right]$, $W_{12}^{*\left(2\right)}=\left[\begin{array}{lll} 100\; & \;0\; & \;0\;\\ 932 & 68 & \;0\;\\ 944 & 56 & \;0\; \end{array}\right]$, $W_{21}^{*\left(2\right)}=\left[\begin{array}{lll} 550 & 450 & \;0\;\\ \;0\; & 35 & 965\\ \;0\; & \;0\; & \;0\; \end{array}\right]$, $W_{12}^{*\left(3\right)}=\left[\begin{array}{lll} \;0\; & 127 & 873\\ \;0\; & \;0\; & 100\\ \;0\; & \;0\; & 100 \end{array}\right]$,$W_{21}^{*\left(3\right)}=\left[\begin{array}{lll} \;0\; & \;0\; & \;0\;\\ 100 & \;0\; & \;0\;\\ 157 & 134 & 709 \end{array}\right]$, $W_{22}^{*\left(1\right)}=\left[\begin{array}{lll} \;0\; & 100 & \;0\;\\ 100 & 100 & \;0\;\\ \;0\; & 100 & \;0\; \end{array}\right]$, $W_{22}^{*\left(2\right)}=\left[\begin{array}{lll} \;0\; & \;0\; & \;0\;\\ \;0\; & \;0\; & \;0\;\\ 100 & \;0\; & \;0\; \end{array}\right]$, $W_{22}^{*\left(3\right)}=W_{22}^{*{\left(2\right)}^{\prime }}$, and $O={\left[0\right]}_{3\times 3}$.

Eq. (3) is used for both ordinary and modified IDW matrix. The difference lies in the matrix entry where $W_{11}^{\left(1\right)}=\left[\begin{array}{lll} \;0\; & 772 & 228\\ 705 & \;0\; & 295\\ 457 & 490 & \;0\; \end{array}\right]$, $W_{12}^{\left(2\right)}=\left[\begin{array}{lll} 100\; & \;0\; & \;0\;\\ 603 & 397 & \;0\;\\ 652 & 348 & \;0\; \end{array}\right]$, $W_{21}^{\left(2\right)}=\left[\begin{array}{lll} 497 & 503 & \;0\;\\ \;0\; & 147 & 853\\ \;0\; & \;0\; & \;0\; \end{array}\right]$, $W_{12}^{\left(3\right)}=\left[\begin{array}{lll} \;0\; & 597 & 403\\ \;0\; & \;0\; & 100\\ \;0\; & \;0\; & 100 \end{array}\right]$, $W_{21}^{\left(3\right)}=\left[\begin{array}{lll} \;0\; & \;0\; & \;0\;\\ 100 & \;0\; & \;0\;\\ 309 & 325 & 366 \end{array}\right]$,$\;W_{22}^{\left(1\right)}=W_{22}^{*\left(1\right)}$,$\;W_{22}^{\left(2\right)}=W_{22}^{*\left(2\right)}$, and $\;W_{22}^{\left(3\right)}=W_{22}^{*\left(3\right)}$.

The modified IDW matrix $\left(W^{*(i)}\right)$ gives some different results compared to ordinary IDW matrix $\left(W^{\left(i\right)}\right)$. The matrix obtained becomes proportional to population size. Meanwhile, there are also italic no change in the weight matrix element. For example, the comparison between $W_{11}^{*\left(1\right)}$ and $W_{11}^{\left(1\right)}$, the weight for location two to one $\left(w_{12}\right)$ is $418\times 10^{-3}$ using the IDW matrix, while the weight for the same location $\left(w_{12}\right)$ is $772\times 10^{-3}\;$ by using the ordinary weight matrix. The more population in a province, the higher the potential for the movement of people. The result is an impact on the potential for transmission of the virus, which will also be even higher.

## 3 Data analysis

The data used are daily cases of 2019-nCoV in six provinces of Java island, which obtained from Task Force for the Acceleration of the Handling 2019-nCoV Republic of Indonesia. The size is 82 days, from March 17^th^ until June 7^th^, 2020. For modeling, the data were divided into two groups; there are 75 and seven observations that are used to measure the fit of the model respectively in parameter estimation of the data (we call it as in-sample) and in predictions (out-sample). Data processing uses R software version 1.2.5033 (r-project.org) by building a peculiar syntax for this case. Figure 3(a) shows the time series plot of each location. Among the six provinces used, Jakarta is the epicenter of the increase of 2019-nCoV with a maximum of 7905 cases. The highest average number of cases added per day is also in Jakarta, 94 cases. In contrast, the smallest is in DIY, which is in the middle of Center Java, only 3 cases. The full descriptive statistics of the daily 2019-nCoV cases in Java are given in Table 3.

**Table 3:** Descriptive statistics of daily cases 2019-nCoV in six provinces. The bold numbers show the highest values in one province compared to other provinces.

	Banten	DKI Jakarta	West Java	Central Java	DIY	East Java
Maximum	71.00	**236.00**	198.00	98.00	16.00	502.00
Total	1031.00	**7905.00**	2375.00	1609.00	244.00	5948.00
Mean	12.42	**95.24**	28.61	19.39	2.94	71.66
Variance	140.88	1998.65	1054.68	357.68	12.33	**8699.13**
St. Dev	11.87	44.71	32.48	18.91	3.51	**93.27**

### 3.1 Modeling data

One of the assumptions in space-time analysis is the correlation among locations. Strong correlation indicates a solid relationship between locations. The correlations were not calculated only at the same time (lag 0), but also with different time lags, depending on the lag when the maximum cross-correlation between locations is reached. Table 4 shows the correlation of daily 2019-nCoV cases among the provinces. The most significant correlation is 0.61 (between Jakarta and Central Java), in which the lag is 13. It means the 2019-nCoV cases in DKI Jakarta at time t give a positive correlation to 2019-nCoV cases in Central Java at time t+13. The smallest correlation is 0.16 (between West Java and Banten), in which the lag is 0. It means the 2019-nCoV cases in West Java gives a positive correlation to 2019-nCoV cases in Banten both at time t. DKI Jakarta gives a significant correlation to other provinces at time t+k, where k is lag of times.

**Table 4:** Correlation of daily cases Co-19 among the provinces. The first and second sub-row of every province respectively represents the correlation at the same time (lag 0), and the correlation at the different time (lag k). Furthermore, the biggest positive correlation and the longest lag were shown respectively by the bold and *italic-bold* writing. Positive correlation shows a unidirectional relationship between two variables, while negative correlation shows the opposite.

Corr. (Lag)	Yi,t + k					
	Banten	DKI Jakarta	West Java	Central Java	DIY	East Java
Banten	1.00 (0);	0.09 (0);	0.15 (0);	-0.17 (0);	-0.05 (0);	0.11 (0);
	0.31 (***15***)	0.26 (5)	-0.27 (12)	-0.28 (9)	0.27 (2)	0.33 (1)
DKI Jakarta	0.09 (0);	1.00 (0);	0.07 (0);	**0.33** (0);	0.21 (0);	0.27 (0);
	-0.36 (10)	0.56 (2)	0.29 (11)	**0.61** (13)	0.25 (11)	0.28 (3)
West Java	0.16 (0);	0.07 (0);	1.00 (0);	0.01 (0);	0.12 (0);	0.00 (0);
	0.16 (0)	0.26 (4)	0.36 (10)	0.47 (1)	0.37 (3)	0.59 (5)
Central Java	-0.17 (0);	0.31 (0);	0.01 (0);	1.00 (0);	0.10 (0);	0.28 (0);
	0.22 (13)	0.31 (0)	0.57 (13)	0.42 (2)	0.47 (14)	0.41 (4)
DIY	-0.05 (0);	0.21 (0);	0.12 (0);	0.10 (0);	1.00 (0);	0.29 (0);
	0.25 (4)	0.38 (3)	0.39 (6)	0.55 (8)	0.24 (9)	0.30 (2)
East Java	0.11 (0);	0.27 (0);	0.00 (0);	0.28 (0);	0.33 (0);	1.00 (0);
	0.27 (6)	0.38 (4)	0.40 (4)	0.56 (9)	0.52 (1)	0.15 (9)

Based on Table 4, most lags with maximum correlations between provinces are long lags (e.g., lag 9, 10, 11, 13, and 15). This is probably caused by two factors, namely rapid test and incubation period (when the patient is first infected/exposed to the virus, so it shows the initial symptoms). Note that a high increase in cases does not mean a virus detected in new people on that day. However, it could be due to the results of tests just came out on that day (stated positive) or the incubation period for the virus. Most people who are entitled to take a rapid test in Indonesia have strictly low-risk or close high-risk contact, or people under monitoring. The rapid test flow is shown in Appendix I. Figure 3(a) also shows the boxplot of the daily 2019-nCoV cases in Java. All provinces have the outliers. Outlier's existence indicates a high case of 2019-nCoV. A significant jump case occurred only once in Banten, DKI Jakarta, and DIY. While in West Java, the surge in cases occurred evenly in early April, May, and late May. The province currently increasing in its 2019-nCoV cases is East Java, mainly at the end of May, with the highest cases per day of 502. While the surge in cases of Central Java only occurred on April. More details of possible causes for a surge in cases (known as outliers) are given in Appendix II.

Another assumption in the GSTAR(1; 1) is stationarity. In Figure 3(a), visually, and based on the Augmented Dickey-Fuller (ADF) test with a significance level of 95%, can indicate that the data plot is not stationary. Stationary test with Augmented Dickey-Fuller (ADF) is a stationary test by determining whether the time series data contains a unit root. The null hypothesis is the unit root exists then the data is not stationary. Test statistics used is $DF=\hat{\delta }/SE\left(\hat{\delta }\right)$, where DF is Dickey-Fuller's value, $\hat{\delta }$ is estimated value and $SE\left(\hat{\delta }\right)$ is the standard error of $\hat{\delta }.$ If the data is not stationary, then do the first differentiation by calculating the difference between observations at time t and observations at time t−1. First differentiation will be subject to the data (see Figure 3(b)), then the stationary data is obtained.

The GSTAR(1; 1) considers first lag in both time and spatial lag. It represents the 2019-nCoV cases on a certain day in a province influenced by the cases in the previous day in that province and nearby provinces. Let $\left\{Z_{t}\right\}$ follows the GSTAR(1; 1) with modified IDW matrix (see Eq. (1)). Since $Z_{t}=Y_{t}-Y_{t-1},$ then

(4) $$\begin{array}{l} Y_{t}-Y_{t-1}={\text{Φ}}_{10}\left(Y_{t-1}-Y_{t-2}\right)+{\text{Φ}}_{11}W^{*}\left(Y_{t-1}-Y_{t-2}\right)+e_{t}\\ Y_{t}=\left({\text{Φ}}_{10}\;+{\text{Φ}}_{11}W^{*}+I\;\right)Y_{t-1}-\left({\text{Φ}}_{10}\;+{\text{Φ}}_{11}W^{*}\right)Y_{t-2}+e_{t} \end{array}$$

where $Z_{t}$ is stationary data and $Y_{t}$ is initial data of daily cases 2019-nCoV at time t., ${\text{Φ}}_{10}$. and ${\text{Φ}}_{11}$. are the autoregressive parameters for time and spatial, I. is identity matrix, $e_{t}$. is noise process at time t., and $W^{*}$. is the modified IDW matrix.

The parameter ${\text{Φ}}_{11}$ are estimated by using Least Square method. Table 5 shows the comparison between parameter estimation using ordinary and modified IDW matrix.

**Table 5:** Parameter of GSTAR(1; 1) model using two kinds of weight matrix (see Eq. (4)). There are some values which show the noticeable differences between using ordinary and modified IDW.

Weight Matrix	Parameter Estimation
Ordinary IDW	${\hat{\text{Φ}}}_{10}=diag\left(-0.516;-0.656;-0.467;-0.595;-0.459;-0.761\right)$
	${\hat{\text{Φ}}}_{11}=\;\text{diag}\left(-0.025;\;0.245;\;-0.253;\;0.412;\;0.016;\;1.105\right)$
Modified IDW	${\hat{\text{Φ}}}_{10}=diag\left(-0.508;-0.662;-0.465;-0.585;-0.457;-0.751\right)$
	${\hat{\text{Φ}}}_{11}=\;\text{diag}\left(0.059;\;0.033;\;-0.144;\;0.067;\;0.025;\;1.227\right)$

The noticeable differences were seen in the spatial autoregressive parameters, for DKI Jakarta and Central Java. By only assuming spatial correlation based on distance, DKI Jakarta gives noticeable and far different parameters compared to the other weight matrixes. Meanwhile, by adding the population ratio factor, the parameter coefficient does not dominate other provinces. It means the population in a province is one of the factors that can increase the daily 2019-nCoV cases. By substituting the parameters to the Eq. (4) and using the modified IDW matrix in Section II, the parameter coefficients for each province is in Table 6.

**Table 6:** Parameter coefficients of GSTAR(1; 1) model using modified IDW matrix.

	Banten [*Y*_1_,_*t*–1_, *Y*_1,*t*–2_]^*t*^	DKI Jakarta [*Y*_2_,_*t*–1_, *Y*_2_,_*t*–2_]^*t*^	West Java [*Y*_3_,_*t*–1_, *Y*_3_,_*t*–2_]^*t*^	Central Java [*Y*_4_,_*t*–1_, *Y*_4,*t*–2_]^*t*^	DIY [*Y*_5_,_*t*–1_, *Y*_5_,_*t*–2_]^*t*^	East Java [*Y*_6_,_*t*–1_, *Y*_6_,_*t*–2_]^*t*^
Y1,t	[0.492, 0.508]	0.021 [1, –1]	0.029 [1, –1]	0.005 [1, –1]	0.001 [1, –1]	0.004 [1, –1]
Y2,t	0.011 [1, –1]	[0.339, 0.662]	0.018 [1, –1]	0.003 [1, –1]	0.001 [1, –1]	0.002 [1, –1]
Y3,t	-0.030 [1, –1]	-0.029 [1, –1]	[0.535, 0.465]	0.046 [1, –1]	0.006 [1, –1]	0.033 [1, –1]
Y4,t	0.006 [1, –1]	0.005 [1, –1]	-0.026 [1, –1]	[0.415, 0.585]	0.014 [1], -1]	0.016 [1, –1]
Y5,t	0.001 [1, –1]	0.001 [1, –1]	0.004 [1, –1]	0.016 [1, –1]	[0.543, 0.457]	0.003 [1, –1]
Y6,t	0.117 [1, –1]	0.099 [1, –1]	0.529 [1, –1]	0.447 [1,–1]	0.084 [1, –1]	[0.249, 0.751]

The GSTAR model obtained can be justified by the results of correlation's number of cases among provinces in Table 4. Provinces that have the maximum correlation in the second time lag, are DKI Jakarta with itself, Central Java with itself, and Yogyakarta with East Java. The number of daily 2019-nCoV cases in DKI Jakarta was influenced by the number of daily cases of 2019-nCoon the previous day by 0.339 and two days before by 0.662 in DKI Jakarta (correlation between DKI Jakarta and itself is 0.56, see Table 4). Other provinces also influence the increase in daily cases of 2019-nCoV in DKI Jakarta, but they are not significant. While other provinces such as Banten, West Java, and Central Java contributed significantly to the increase in daily cases of 2019-nCoV in East Java, respectively, by 0.117; 0.529 and 0.447. The increasing 2019-nCoV cases in a province is more influenced by the number of 2019-nCoV cases in that province itself except for East Java. This is possibly due to government policies that forbid residents in one province to move to another province, namely large-scale social restrictions.

**Figure 4:**
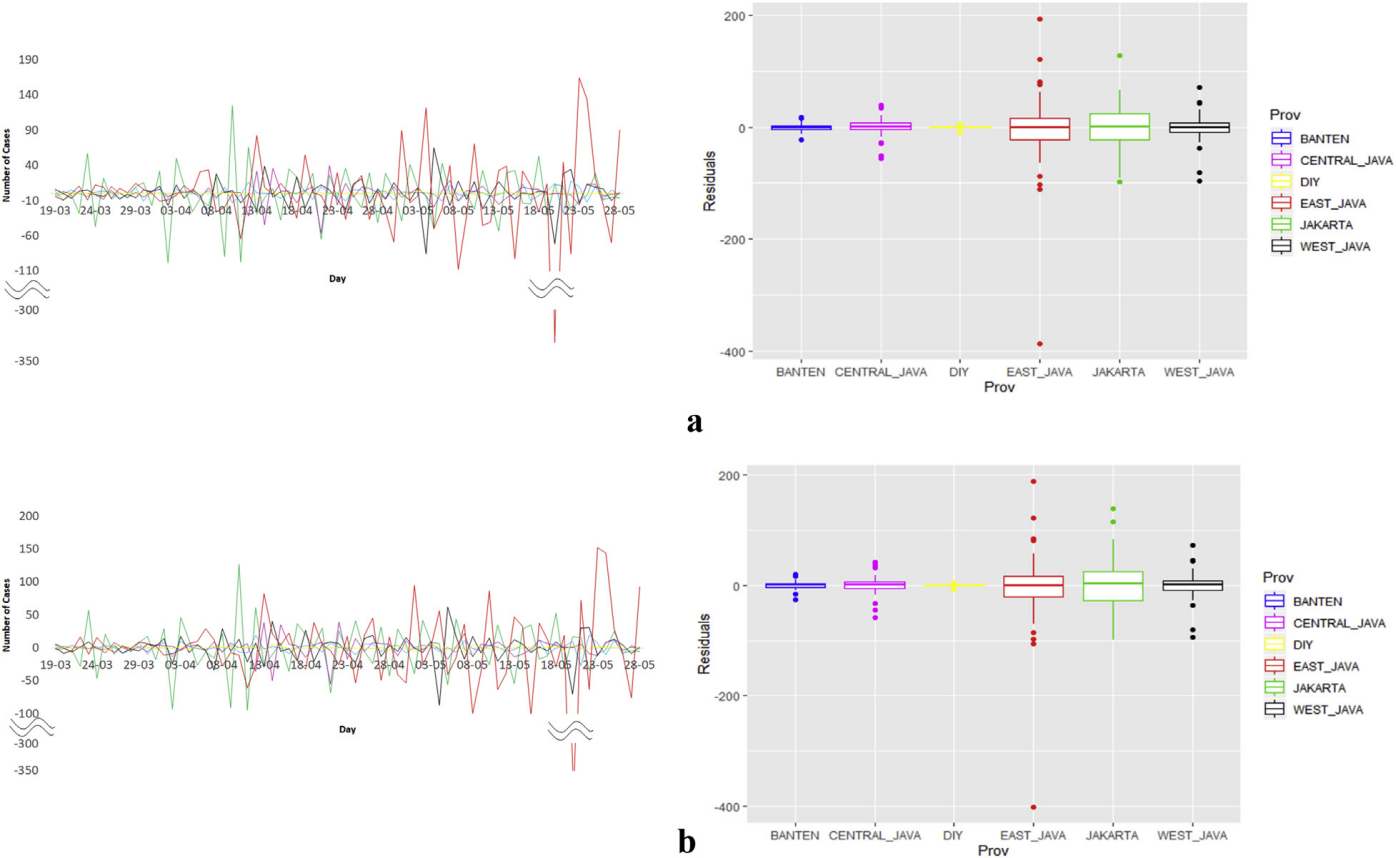
Residual plot and boxplot based on GSTAR(1; 1) using (a) modified weight matrix and (b) IDW matrix. Residual in East Java is still fluctuating, different from Banten, DIY, and Central Java.

Residual checking is needed in spatio-temporal modeling to see the normality and independence of residuals. Those are the underlying assumptions of the model, called the white noise residual. Fluctuating residuals indicate a significant difference between observations and fitted values based on the GSTAR(1; 1). For testing the assumptions of normality and residuals independence, Kolmogorov-Smirnov (K–S) and Ljung-Box test are used respectively. The null hypotheses are residuals follow normal distribution (K–S test) and independence (Ljung-Box test). All p-values obtained for both types of hypotheses are less than 0.1% (null hypotheses are rejected), except for normality test of Central Java residuals. For both modified and ordinary IDW, null hypotheses are not rejected if α is less than 3%. Figure 4 shows the time series plot and boxplot of the residuals using the GSTAR(1; 1). From the residuals boxplot of the two GSTAR(1; 1), they did not show noticeable differences related to the number of outliers detected. The only difference found is in Banten province, which the modified IDW matrix can reduce one outlier of the residual compared to the GSTAR(1; 1) using the ordinary IDW matrix. It shows another good point of modified compared to ordinary IDW matrix.

### 3.2 Forecasting

**Figure 5:**
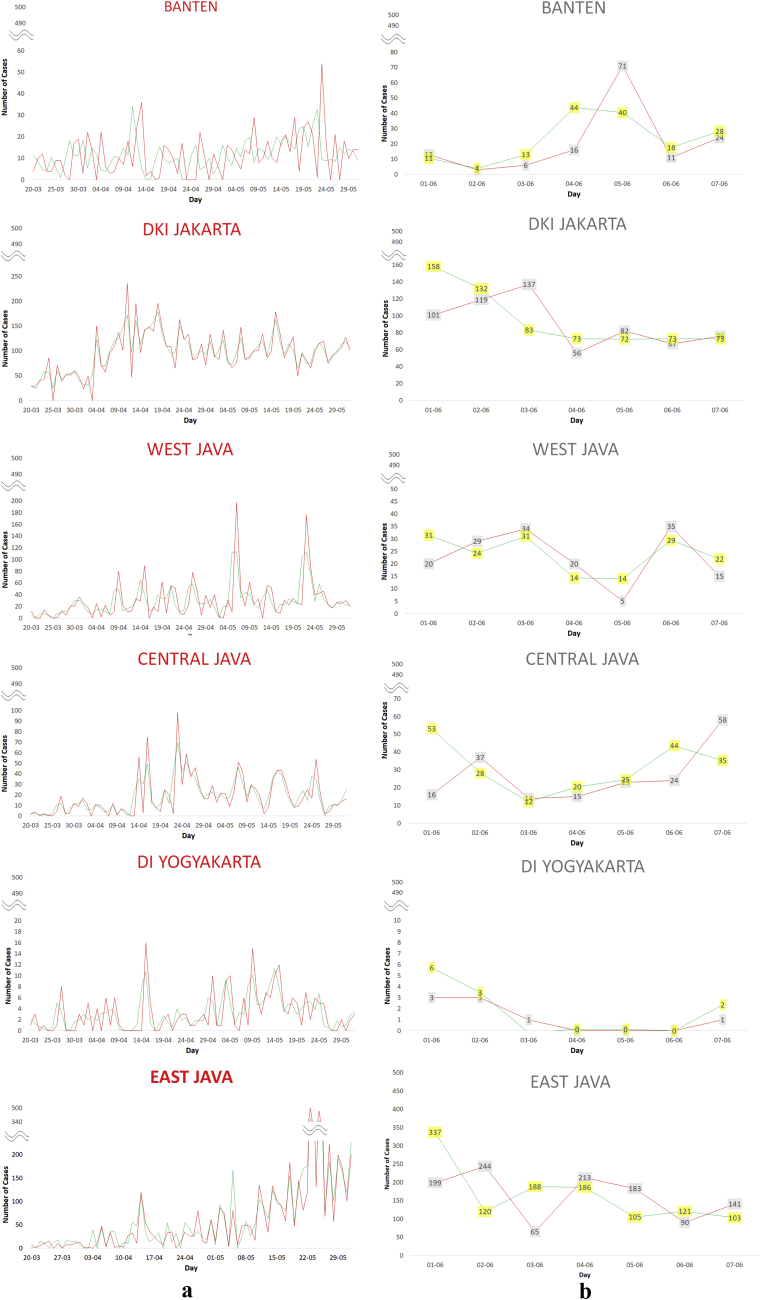
(a) Testing the model for in-sample data by updating the data using the entire observations, and (b) Testing the model for out-sample data by updating the prediction just using the error. The red line is for observation, while the green line is for fitted values using GSTAR(1; 1) model with modified IDW matrix.

**Figure 6:**
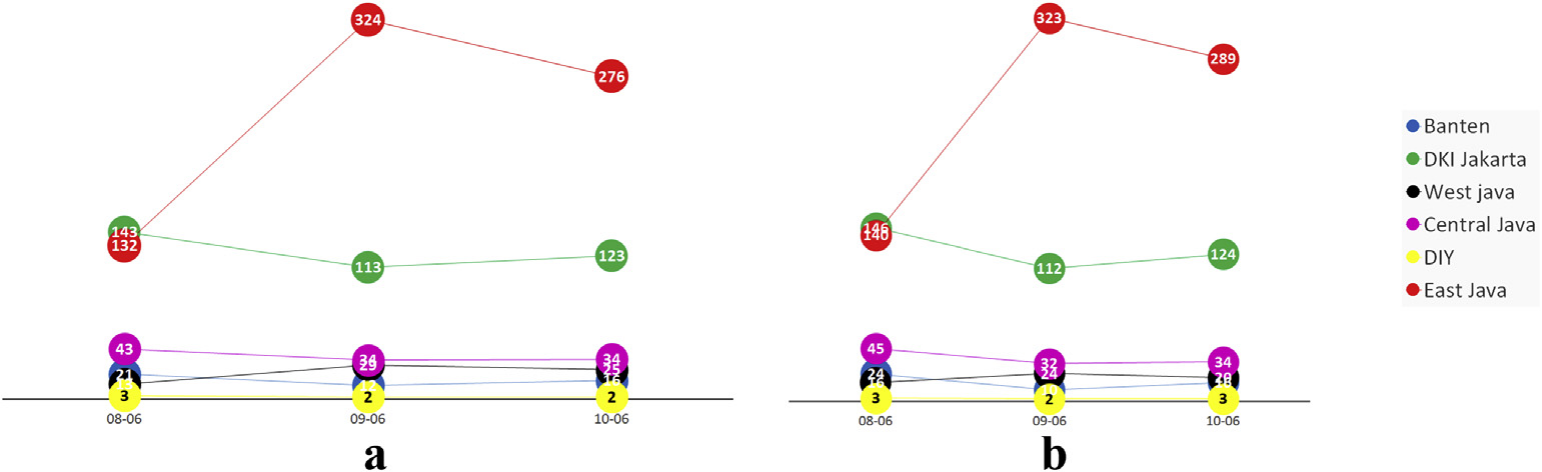
Forecasting three times using GSTAR(1; 1) with (a) modified and (b) ordinary IDW matrix. Prediction results in Banten, DIY, West and Central Java using both (a) and (b) are tend to be flat. Meanwhile, East Java and DKI Jakarta show fluctuating prediction results. East Java is ranked first in the number of additional cases of 2019-nCoV, ahead of DKI Jakarta as the province with the most cases in Indonesia.

The primary purpose of modeling data is forecasting. Table 7 gives the Mean Square Residual (MSR) to see the goodness of fit of the model while estimating (in-sample) and forecasting (out-sample). East Java gives the most noticeable difference between in-sample and out-sample (see also Figure 4(b)). This is due to the provincial government conducting extensive rapid tests on its population. Also, a new cluster was detected identified in Surabaya, East Java, after at least 36 employees of the cigarette factory “Sampoerna” is positively diagnosed in May, 8th 2020 (see Appendix II). Figure 5 shows the in-sample (a) and out-sample (b) results using GSTAR(1; 1) with IDW matrix. In the in-sample data, fitted values obtained by entering all data into the model. In the out-sample data, the model is used to predict without updating each observation, but to update it based on the prediction error (Cryer and Chan, 2008). The comparison is executed between the original and modified IDW matrix results. In terms of average residual, modifying the IDW matrix gives good results, both for in-sample and out-sample data (except Banten) although it is just slightly different Of the two weight matrices, the GSTAR(1; 1) gives better results in predictions, as evidenced by the mean squared residuals of all provinces are smaller for both types of data. Therefore, the GSTAR (1; 1) can be used to predict on June 8^th^, 9^th^, and 10^th^, as shown in Figure 6.

**Table 7:** MSR for in-sample (bold and first row) and out-sample (second row) data using GSTAR(1; 1) model with ordinary and modified IDW matrix (modified (ordinary)).

Banten	DKI Jakarta	West Java	Central Java	DIY	East Java	Av. Residual
**55.56 (53.74)**	**1296.19 (1307.27)**	**406.82 (415.80)**	**189.68 (202.60)**	**5.00 (4.81)**	**4228.56 (4712.51)**	**1030.3 (1116.1)**
251.98 (236.65)	357.63 (461.18)	149.00 (157.82)	113.45 (165.72)	2.47 (1.79)	1920.14 (1775.03)	465.78 (466.37)

## 4 Conclusions and remarks

Government policies and readiness are crucial to addressing 2019-nCoV impacts. Large-scale social restriction polices can reduce 2019-nCoV cases because they can reduce the movement of people from one province to another. That policy's effect can be seen from the results of the GSTAR(1; 1) model obtained (see Table 5), the increasing of 2019-nCoV cases in a province is more influenced by the 2019-nCoV cases developed in that province. If the policy easing is carried out, the influence of other provinces will be more significant, resulting in a surge in the 2019-nCoV case and the addition of a new epicenter. Based on our result, East Java is the new epicenter. Further, the massive rapid tests are the key to accelerate the end of the pandemic. The more people identified, the faster treatment will be given, and as a result, we can control the transmission of the virus.

Modifying the IDW matrix by adding the population ratio produces a more representative GSTAR(1; 1) model than ordinary IDW matrix. This is also characterized by a smaller average residual value when compared to the ordinary IDW matrix (see Table 7). Other provinces besides East Java are not affected by the 2019-nCoV daily case in other provinces. However, East Java is the only province with a significant increase in the daily cases of 2019-nCoV influenced by other provinces, with parameter coefficients are 0.117; 0.099; 0.529; 0.447; 0.084 respectively for Banten; West Java; Central Java; DIY. This is caused by one of the habits of the East Javanese people to migrate to other areas to get a better livelihood. This habit has been started since the old order government in the transmigration program was motivated by equitable development. This movement was a significant factor in the enormous influence of daily cases from other provinces to East Java. Until now, East Java has become the province with the most significant addition of 2019-nCoV daily cases. In terms of forecasting, the GSTAR(1; 1) model using modified is good enough for the prediction of 2019-nCoV increment cases in DKI Jakarta, West Java and East Java. The weight matrix is used based on the distance of a train that moves from one station in provinces A to B. In this weight matrix; it is not assumed that many passengers can be accommodated on the train. It can be added, on average, how many people move from one province to another by train.

## 5 Future research

As the number of observations increase, the weight matrix may change. Therefore, the weight matrix should be considered as random matrix which has probability distribution. The idea is to build small interval for each element of weight matrix with its expected values are the obtained (current) weight matrix, then generate random numbers within those intervals. All possibilities and combination values will be considered as the realization of random variables. From those values, the combination which give the best result in prediction or estimation will be the new weight matrix. Furthermore, due to some existences of extreme values, known as outliers, it is recommended to apply GSTAR model with outlier factors. If the existence of outliers is ignored, the model has a high possibility to have non-normality and correlated residuals.

## Declarations

### Author contribution statement

U. S. Pasaribu, S. W. Indratno: Conceived and designed the experiments; Analyzed and interpreted the data; Contributed reagents, materials, analysis tools or data; Wrote the paper.

U. Mukhaiyar: Conceived and designed the experiments; Performed the experiments; Analyzed and interpreted the data; Contributed reagents, materials, analysis tools or data; Wrote the paper.

N. M. Huda: Conceived and designed the experiments; Performed the experiments; Analyzed and interpreted the data; Wrote the paper.

K. N. Sari: Conceived and designed the experiments; Analyzed and interpreted the data; Wrote the paper.

### Funding statement

This work was supported by the RISTEK-DIKTI grant, Fundamental Research, 2020.

### Data availability statement

The data was available online daily at https://covid19.go.id/peta-sebaran.

### Declaration of interests statement

The authors declare no conflict of interest.

### Additional information

No additional information is available for this paper.

## Figures and Tables

**Figure:**
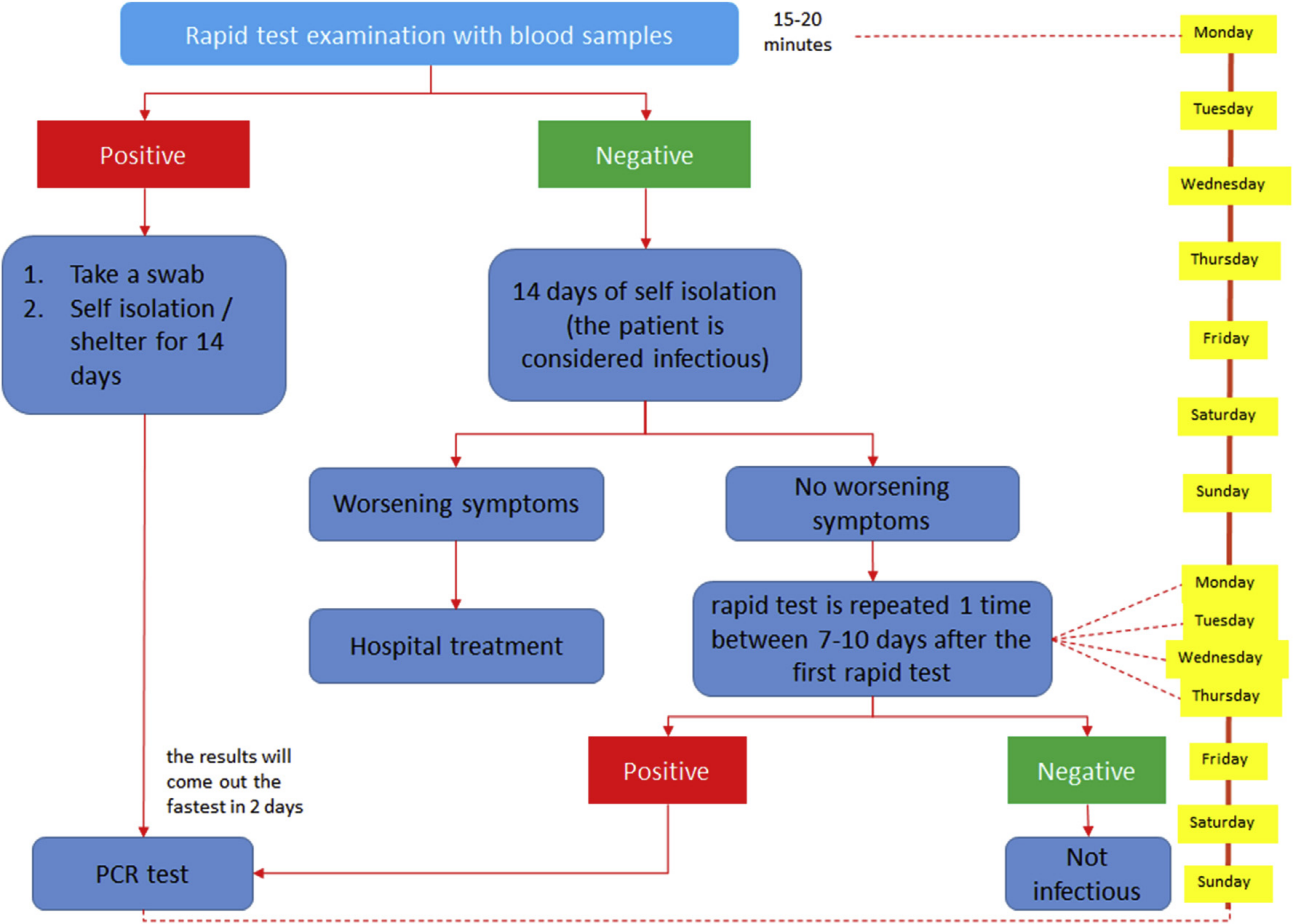


**Table:** 

Provinces	Date	Cases	Additional Information
Banten	May 21^st^	54	PDP and ODP patients today jumped from Saturday (May 9^th^). Spokesperson of the Task Force to Accelerate Handling of 2019-nCoV, Ati Pramudji Hastuti, said the surge occurred because the South Tangerang Health Office did not report to Banten.
DKI Jakarta	April 9^th^	236	On March 25^th^, The Indonesian Ministry of Transportation discouraged everyone from travelling home for the Eid al-Fitr holiday to contain the spread of the virus. On April 2^nd^, Indonesia's Justice and Human Rights Ministry will release more than 30,000 prisoners to avoid mass coronavirus infections in Indonesia's overcrowded jails.
West Java	April 13^th^	90	April 3^rd^, West Java Provincial Government has conducted rapid tests by way of door to door to residents' homes until the implementation of the rapid test drive-thru.
	May 4^th^	198	On April 21^st^, Joko Widodo announced his decision to ban the *Idul Fitri mudik* (exodus) starting from April 24 to curb the spread of 2019-nCoV ahead of Ramadan.
	May 20^th^	176	On May 7^th^, The Ministry of Transportation allows intercity transportation to resume operation when transporting passengers for essential business, repatriation, or governmental purposes, but not “*mudik*”
	May 21^st^	86	
Central Java	April 12^nd^	56	On April 7^th^, Massive rapid test or rapid test to detect the corona virus or 2019-nCoV was held in all districts and cities in Central Java.
	April 14^th^	75	
	April 21^st^	98	On April 15^th^, Central Java Provincial Health Service (Central Java) will re-distribute the corona virus rapid detection tool which is a rapid diagnostic test (RDT) to the regency and city areas.
DIY	April 13^rd^	16	The City Government of Yogyakarta recorded 1,022 newcomers, including city residents who had just returned from outside the area, into the city of Yogyakarta in early April 2020.
East Java	May 21^st^	502	On May 7^th^, The Ministry of Transportation allows intercity transportation to resume operation when transporting passengers for essential business, repatriation, or governmental purposes, but not “*mudik*”. On May 8^th^, A new 2019-nCoV cluster is identified in Surabaya, East Java after at least 36 employees of the cigarette factory *Sampoerna* is positively diagnosed.
	May 23^rd^	466	
	May 25^th^	223	
	May 30^th^	199	
	May 31^st^	244	

## Appendices

I. Rapid test flow. If someone takes a rapid test on Monday, then someone's results are said to be positive co-19 most quickly obtained after 13 days (with the following simulation: on the first test, the results are negative and on the second test the results are positive).

II. Information which is the cause of increasing 2019-nCoV cases in Java (known as outlier). Today's surge in cases is caused by what happened a few days ago (see Table 4). Most are caused by massive rapid tests (see the flow of rapid tests and their simulations in Appendix I) and government policies.
